# PCDHB17P/miR-145-3p/MELK/NF-κB Feedback Loop Promotes Metastasis and Angiogenesis of Breast Cancer

**DOI:** 10.3389/fonc.2021.660307

**Published:** 2021-07-19

**Authors:** Li Zhu, Yan-Jun Zhang, Bin Wang, Li Yang, Yi-Qiong Zheng, Lin-De Sun, Lin Tian, Tao Chen, Jian-Dong Wang

**Affiliations:** ^1^ Department of General Surgery, The First Medical Center of Peoples Liberation Army General Hospital, Beijing, China; ^2^ Department of General Surgery, The Second Medical Center of Peoples Liberation Army General Hospital, Beijing, China; ^3^ Department of General Surgery, Beijing Shunyi Hospital, Beijing, China; ^4^ Department of Cardiology, The First Medical Center of Peoples Liberation Army General Hospital, Beijing, China

**Keywords:** PCDHB17P, metastasis, angiogenesis, breast cancer abstract, miR-145-3p

## Abstract

Breast cancer is one of the most common life-threatening cancers, mainly because of its aggressiveness and metastasis. Accumulating evidence indicates that long non-coding RNAs (lncRNAs) participate in the development and progression of breast cancer. Nevertheless, the function and expression level of lncRNAs in breast cancer are still not fully understood. Here, we demonstrated that lncRNA PCDHB17P was up-expressed in human breast cancer tissues and cell lines. Knockdown of PCDHB17P remarkably suppressed migration and invasion, as well as tube formation ability of breast cancer cells. MiR-145-3p was significantly decreased in breast cancer samples, which was negatively correlated to the expression of PCDHB17P. In addition, we identified that MELK was a direct target gene of miR-145-3p, which was higher expressed in breast cancer tissues than that in adjacent normal tissues. Mechanistic investigation indicated that PCDHB17P acted as a cancer-promoting competing endogenous RNA (ceRNA) by binding miR-145-3p and upregulating MELK. Interestingly, MELK could in turn increase the promoter activity and expression of PCDHB17P *via* NF-κB, thus forming a positive feedback loop that drives the metastasis and angiogenesis of breast cancer. Overall, the results demonstrated that the constitutive activation of PCDHB17P/miR-145-3p/MELK/NF-κB feedback loop promotes the metastasis and angiogenesis of breast cancer, suggesting that this lncRNA might be a promising prognostic biomarker and therapeutic target for breast cancer.

## Introduction

According to global cancer statistics in 2018, breast cancer is the second commonly diagnosed cancer (11.6% of the total cases), and ranks one of the most commonly diagnosed cancers and the primary cause of cancer-related death in females worldwide (1). Despite advances in diagnosis and treatment, the 5-year survival rate for patients with advanced breast cancer is still low (2, 3). In fact, it is estimated that more than 90% of patients died of relapse and metastasis (2). Metastasis relies on an array of processes, and angiogenesis, which begins with the sprouting of new blood vessels from preexisting vascular network, is one of the key processes that mediates metastasis (4). However, mechanisms that investigate into promising molecular biomarkers that could predict the risk of metastasis, and angiogenesis of breast cancer are still in its infancy. Studies have shown that tumor angiogenesis activates the proliferation, invasion, and metastasis of cancer cells, thus playing an important role in the initiation and development of solid tumors, including breast cancer (5, 6). It is well known that the more pathological angiogenesis occurs, the more cancer cells could migrate into the circulatory system and achieve distant metastasis (7). Therefore, inhibiting angiogenesis might be an attractive strategy for cancer therapy, which is of great importance in clinic treatment of breast cancer.

As we all know, approximately 98% of the genomes do not further translate into proteins, which are regarded as non-coding RNAs (ncRNAs). Among these ncRNAs, long noncoding RNAs (lncRNAs) are transcripts longer than 200 nucleotides, and play an essential regulatory role in various tumor (8). Accumulating studies have reported that lncRNAs are associated with metastasis and angiogenesis of cancers. For instance, LINC00673 was involved in the regulation of breast cancer cell metastasis by regulating the epithelial-mesenchymal transition (EMT) (9). HITT was commonly decreased in multiple human cancers, which regulated feedback loop with HIF-1α to modulate angiogenesis and tumor growth of colon cancer (10). SOX9-AS1 promoted the proliferation, migration, and invasion of HCC cells through the Wnt/β-catenin pathway (11). Further researches on the functions of lncRNA could contribute to deeper understanding of cancer biology and provide new opportunities for the diagnosis and treatment of cancer (12). In our study, based on the analysis of breast cancer TCGA datasets, we identified a breast cancer-related lncRNA PCDHB17P, which was apparently highly expressed in breast cancer tissues compared with the normal tissues. In this basis, we wondered whether PCDHB17P was implicated in breast cancer development.

It has been largely reported that lncRNAs act as ceRNAs to regulate microRNAs (miRNAs) with shared response elements and dysregulate tumor oncogenes and suppressor genes (13). Chondrosarcoma cell-derived exosomes carry lncRNA RAMP2-AS1, which acted as a ceRNA of miR-2355-5p to regulate VEGFR2 expression, thereby positively regulating the angiogenic ability of HUVECs (14). MiR-3064-5p exerted an antiangiogenic role by targeting the FOXA1/CD24/Src pathway in hepatocellular carcinoma (HCC) but oncogenic lncRNA MALAT1 acted as a ceRNA to sponge miR-3064-5p (15).

In the present study, we investigated the role of PCDHB17P in breast cancer and found its promotion effect on migration and tumor-related angiogenesis. Using online database, we found potential binding sites between PCDHB17P and miR-145-3p. In addition, MELK was predicted as the downstream target gene of mir-145-3p, which further elevated the expression of NF-κB, leading to the transcription of PCDHB17P, VEGFA, and FGF2. Our study revealed a PCDHB17P/miR-145-3p/MELK/NF-kB feedback loop network in breast cancer metastasis and angiogenesis, which gives new insights into how PCDHB17P facilitates the development of breast cancer and might provide new targets for breast cancer treatment.

## Materials and Methods

### Patients and Specimens

24 paired breast cancer tissues and adjacent normal tissues were collected from The First Medical Center of Peoples Liberation Army General Hospital. All participants provided their written informed consent. Tissue samples were frozen in liquid nitrogen, stored at −80°C till RNAs were extracted.

### Cell Lines and Culture Conditions

All human breast cancer cell lines, obtained from the Institute of Biochemistry and Cell Biology of the Chinese Academy of Sciences (Shanghai, China), were cultured in RPMI medium 1640 (GIBCO, Grand Island, USA) with 1% penicillin/streptomycin and 10% FBS. The cells were incubated at 37°C with 5% CO_2_ in a humidified incubator.

### Fluorescence In Situ Hybridization

MDA-MB-231 and MCF-7 cells were fixed with 4% paraformaldehyde at room temperature for 15 min, and 0.5% TritonX-100 was used for permeabilization at 4°C for 20 min. Then, cells were incubated with the FISH probe of PCDHB17P at 55°C overnight. After hybridization, the slides were subjected to washing and dehydration. DAPI was performed to counterstain nuclei. The cells were captured using a fluorescence microscope (Olympus, Tokyo, Japan) and were merged using Adobe Photoshop 6.0 software.

### Cell Transfection

According to the manufacturer’s protocol, MDA-MB-231 and MCF-7 cells were severally transfected with each of the following by utilizing Lipotransfectamine 3000(Invitrogen, California, USA). PCDHB17P-specific short hairpin RNA (sh-PCDHB17P-1, sh-PCDHB17P-2) and negative control (sh-NC) were produced by RiboBio (Guangzhou, China) (**Supplementary Table 1**), along with pcDNA3.1 vector containing PCDHB17P and empty vectors, were all purchased from GenePharma (GenePharma, Shanghai, China). Si-MELK, MiR-145-3p mimics, miR-145-3p inhibitors, and their corresponding miR-NCs were synthesized by RiboBio (Guangzhou, China).

### Quantitative Real-Time PCR Analysis

Total RNA was extracted from tissue specimens or cells using TRIzol reagent (Invitrogen, California, USA). The nuclear RNA and cytoplasmic RNA were separated by Purification Kit (Norgen, Thorold, Canada). Complementary DNA (cDNA) was synthesized with PrimeScript RT Reagent Kit (TaKaRa, Tokyo, Japan). The RT-PCR was accomplished using SYBR green real-time PCR kit (TaKaRa, Tokyo, Japan). GAPDH and U6 expression were, respectively, utilized as the endogenous control for mRNA/lncRNA and miRNA. Gene expression levels were quantified through 2^−ΔΔCt^ method. The primer sequences used in RT-qPCR are listed in **Supplementary Table 2**.

### HUVEC Tube Formation Assay

BD Matrigel™ Matrix was diluted with the serum-free DMEM medium at a ratio of 1:1. Then, 200 μl of mixture was added onto 24-well plates and polymerized for 1 h at 37°C. HUVEC suspension was added into the solidified gel. After 6 h, tube formation was observed and captured with microscopy.

### Wound Healing Assay

The cells were seeded in 6-well plates and incubated at 37°C until the cells reached 90% confluence. Briefly, cell layers were scratched by 200 μl sterile pipette tip and cells were then maintained in DMEM at 37°C and 5% CO_2_. Cell migration was observed and calculated at 0 h and 24 h with an invert microscope.

### Western Blotting Analysis

The cells was collected and dissolved in RIPA lysis buffer (Beyotime, Jiangsu, China) at 4°C, the extracted proteins were separated by SDS-polyacrylamide gel (10%). After electrophoresis, proteins were transferred to polyvinylidene fluoride (PVDF) membranes (Millipore, Massachusetts, USA). Then, PVDF membranes were blocked with 5% skimmed milk for 2 h with gentle shaking, and incubated overnight at 4°C with the following primary detection antibodies: anti-MELK (1:2000, Abcam, Cambridge, England), anti-p65 (1:2000, CST, Boston, USA), anti-p-P65 (1:1000, Abcam, Cambridge, England), anti-IKKβ(1:2000, CST, Boston, USA), and anti-p-IKKβ(1:2000, CST, Boston, USA). The following day, the membrane was washed and incubated with horseradish peroxidase-conjugated secondary antibody (goat anti-rabbit, 1:5000; Wanleibio, Shenyang, Liaoning, China) for 1 h, and the proteins were detected using BeyoECLPlus (Beyotime, Jiangsu, China). GAPDH was used as an internal reference, the relative expression of individual protein was calculated by the ratio of the gray value of the target protein to the internal reference band.

### RNA Immunoprecipitation

The EZMagna RIP Kit (Millipore, Bedford, USA) was used for RIP according to the manufacturer’s protocol. The cells were lysed and incubated in an ice bath with protein A magnetic beads, and centrifuged to collect the supernatant. The cell extract was co-precipitated by incubation with the antibody. The magnetic beads were washed and re-suspended in RIP Wash Buffer. Then, added appropriate antibodies to each group. The magnetic bead-antibody complex and the cell extract were incubated overnight at 4°C. After that, the magnetic bead-protein complex was harvested. The sample was digested with proteinase K to extract RNA for subsequent PCR detection.

### Transwell Assay

Transfected cells were collected with serum-free medium. Approximately 20,000 cells were added into the upper chamber of a transwell (Corning, New York, USA) with or without Matrigel (BD, New Jersey, USA) to evaluate invasion or migration functions. Medium with 15% FBS was placed to the lower chamber. 48 h later, cells that had invaded were fixed by 4% paraformaldehyde and dyed utilizing crystal violet (Beyotime, Jiangsu, China). Cells were observed and captured with microscopy.

### Luciferase Reporter Assay

The sequences of PCDHB17P or MELK 3′-UTR containing the putative binding sites of miR-145-3p were subcloned into a pGL3 Dual-luciferase vector (Promega, Madison, USA). The luciferase reporter plasmids were co-transfected into MDA-MB-231 and MCF-7 cells with miR-145-3p mimics or MELK overexpression (OE) and the negative control. 48 h after transfection, luciferase signals were measured with the Dual Luciferase Reporter Assay System (Promega, Madison, USA) according to the manufacturer’s instructions.

### Chromatin Immunoprecipitation

The ChIP Assay Kit (Millipore, Bedford, USA) was used for ChIP according to the manufacturer’s protocol. Briefly, the cells were collected and sonicated to generate chromatin samples with average fragment sizes of 100 to 500 bp. Then, immunoprecipitated with anti-NF-kB (Abcam, Cambridge, England) or anti-IgG (Abcam, Cambridge, England) antibodies at 4°C overnight. The immunoprecipitated DNA was eluted and purified for the subsequent qPCR analysis.

### Immunohistochemistry

The tissue samples were placed into citrate buffer and heated in a microwave oven. Slides were then incubated with anti-VEGFA (1:100, Abcam, Cambridge, England), anti-MELK (1:500, Abcam, Cambridge, England), or anti-CD33(1:100, Abcam, Cambridge, England) antibody at room temperature for 1 h. Following washing, each section was briefly counterstained using a catalyzed sign amplification system kit (Dako code k5007). The images were captured under the same conditions.

### *In Vivo* Nude Mouse Models

Female BALB/c nude mice (aged 5 weeks) was bought from Guangdong Medical Laboratory Animal Center, and kept under specific pathogen-free conditions. 5 × 10^6^ transfected breast cells were injected subcutaneously into the flank of nude mice, 6 weeks later, the tumors were isolated, and subjected to Immunohistochemistry. For the lung metastasis model, 1 × 10 ^6^ cells were injected into the tail veins of the mice. After 4 weeks, the mice were killed and the lung tissues were fixed and paraffin-embedded for hematoxylin and eosin (H&E) staining.

### Statistical Analysis

Data were analyzed by GraphPad Prism 6.0 and shown as mean ± standard deviations, unless otherwise specified. Differences between groups were analyzed by unpaired Student’s *t*-test between two groups and one-way ANOVA followed by Tukey comparison test in more than three groups. Statistical significance was defined as ^*^ *p* ≤ 0.05, ^**^ *p* < 0.005, and ^***^ *p* < 0.001, respectively.

## Results

### PCDHB17P is Specifically Up-Regulated in Breast Cancer and Predicts Poor Survival

**Figure 1:**
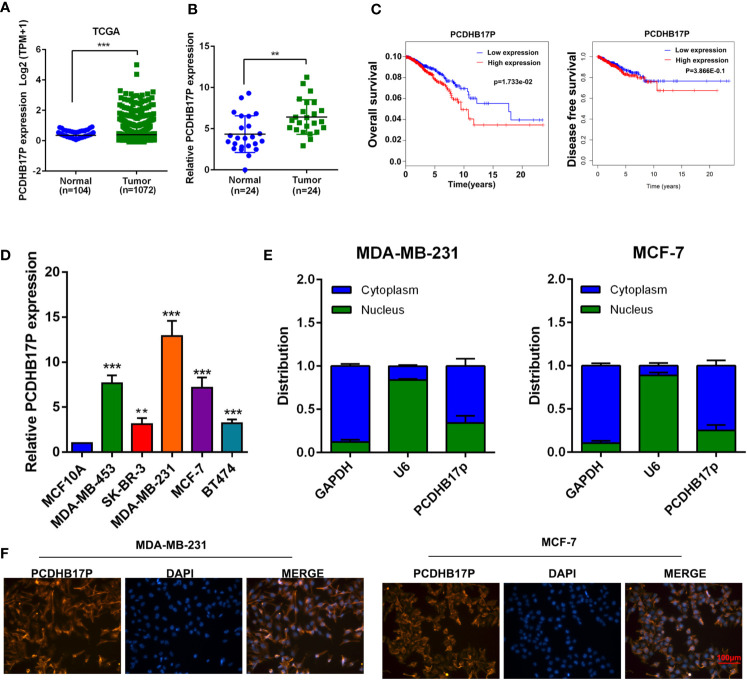
PCDHB17P is specifically up-regulated in breast cancer **(A)** Expression level of PCDHB17P in breast cancer samples and normal tissues from TCGA database. **(B)** The expression of PCDHB17P were detected in breast cancer samples and normal tissues by RT-qPCR. **(C)** Kaplan-Meier analysis of the correlation between PCDHB17P expression and overall survival/disease free survival in breast cancer TCGA dataset. Left: overall survival, Right: disease free survival **(D)** The expression of PCDHB17P were detected in breast cancer cells by RT-qPCR. **(E)** RT-qPCR assay of subcellular PCDHB17P expression in the nucleus and cytoplasm of MDA-MB-231 and MCF-7 cells. GAPDH and U6 were used as endogenous controls. **(F)** Subcellular localization of breast cancer cells detected by RNA-FISH. PCDHB17P is stained red and nuclei are stained blue (DAPI). ^**^ *P* < 0.01, ^***^ *P* < 0.001.

First, we examined the expression file of lncRNAs in breast cancer. Analysis based on the TCGA dataset presented the up-regulation of PCDHB17P in breast cancer samples (**Figure 1A**). Additionally, PCDHB17P expression was elevated in frozen breast cancer tissues compared with that in the adjacent normal tissues by RT-qPCR (**Figure 1B**). Kaplan-Meier survival analysis indicated that increased PCDHB17P expression in breast cancer was significantly associated with a lower rate of overall survival and disease-free survival (**Figure 1C**). Moreover, the expression level of PCDHB17P was up-regulated in breast cancer cell lines than in the mammary epithelial cell, especially in MDA-MB-231 and MCF-7 cells (**Figure 1D**). To elucidate whether PCDHB17P is involved in the breast cancer development, we analyzed the correlation of PCDHB17P expression with clinical pathology factors. There was a significant correlation between PCDHB17P expression and age or lymph node metastasis (**Supplementary Table 3**). Subcellular fractionation analysis-verified PCDHB17P was localized mainly in the cytoplasm of breast cancer cells (**Figure 1E**). FISH staining further confirmed the PCDHB17P fluorescence intensity in the cytoplasm of breast cancer cells (**Figure 1F**). Based on these data, we speculated that PCDHB17P might play a role in breast cancer development.

### PCDHB17P Promotes Metastasis of Breast Cancer Cells

**Figure 2:**
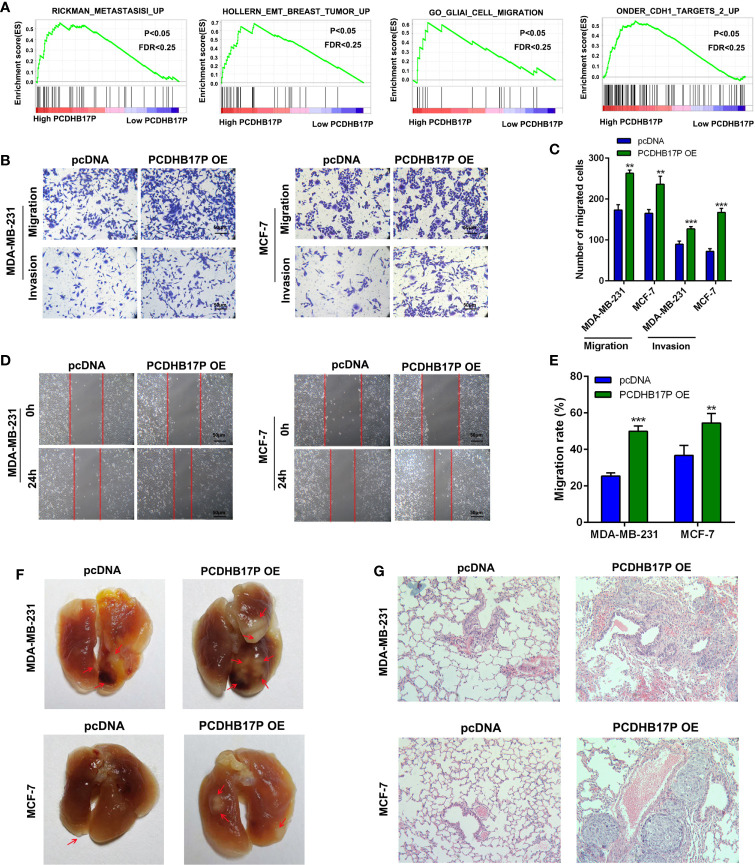
PCDHB17P promotes metastasis of Breast cancer cells **(A)** GSEA revealed that PCDHB17P expression was related to metastasis in breast cancer. **(B–E)** Influence of PCDHB17P overexpression on cell migration and invasion of MDA-MB-231 and MCF-7 cells by the transwell and wound healing assays. **(F, G)** MDA-MB-231 and MCF-7 cells stably overexpressing PCDHB17P were injected into the tail vein of mice to construct a lung metastasis model. Representative images of macroscopic metastatic nodules on lung and HE staining results. ^**^ *P* < 0.01, ^***^ *P* < 0.001.

We next referred to gene set enrichment assay (GSEA) and found that PCDHB17P expression was related to cell metastasis and EMT (**Figure 2A**). To evaluate the influence of PCDHB17P on breast cancer cells metastasis, we upregulated PCDHB17P expressing with the pcDNA-PCDHB17P plasmid and knocked down PCDHB17P expression in MDA-MB-231 and MCF-7 cells with shRNAs against PCDHB17P (sh-PCDHB17P-1 and sh-PCDHB17P-2) (**Supplementary Figure 1A**). Transwell and Wound Healing assays demonstrated that breast cancer cells were enhanced by PCDHB17P overexpression while weakened by PCDHB17P knockdown (**Figures 2B–E**, **Supplementary Figures 1B–D**). The lung metastasis model showed that more microscopic metastatic nodules were formed in lungs of PCDHB17P overexpression group (**Figures 2F, G**). The above mentioned data suggested that PCDHB17P promoted breast cancer cells migration, invasion, and metastasis.

### PCDHB17P Promotes the Angiogenesis of Breast Cancer Cells

**Figure 3:**
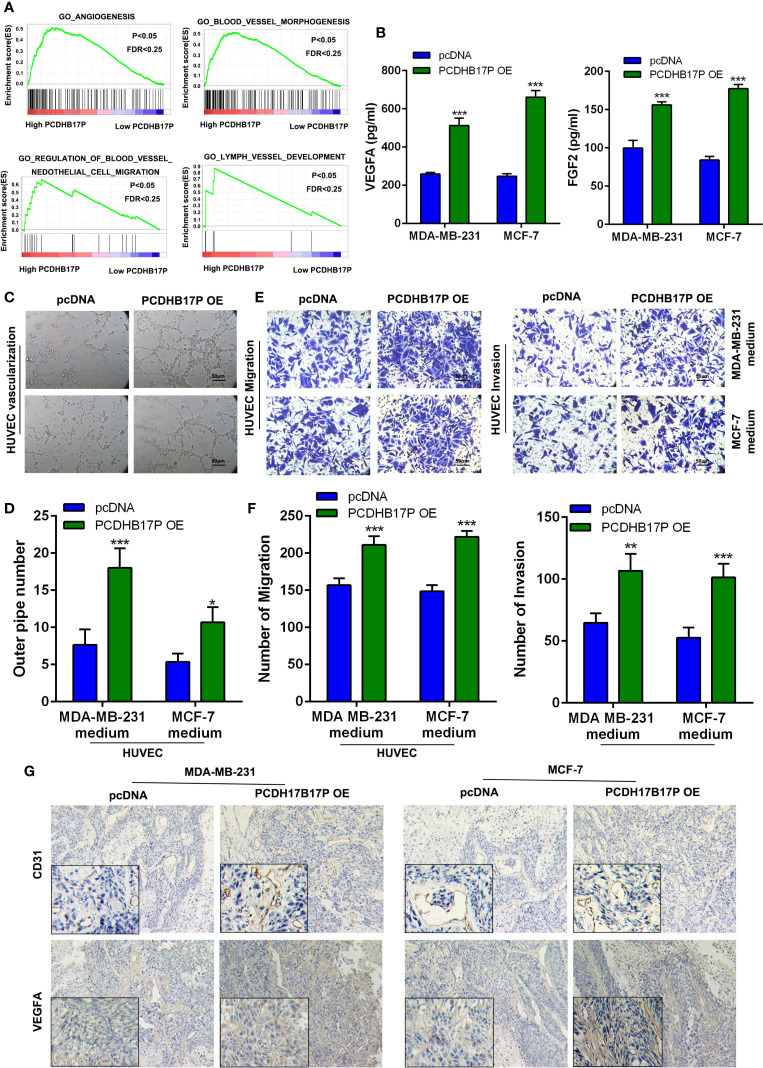
PCDHB17P promotes angiogenesis of BRCA cells **(A)** GSEA revealed that PCDHB17P was related to angiogenesis in breast cancer. **(B)** The protein level of VEGFA and FGF2 in CM by ELISA assays. **(C, D)** Tube formation of HUVEC cell was detected and results were expressed as number of branches. **(E, F)** HUVEC cell migration and invasion were determined by transwell assay. **(G)** The expression of VEGFA and CD31 in the xenografts was examined by IHC. ^*^ *P* < 0.05, ^**^ *P* < 0.01, ^***^ *P* < 0.001.

To further explore the function of PCDHB17P in breast cancer, we referred to GSEA and found that PCDHB17P expression was related to angiogenesis (**Figure 3A**). We detected the expressed of angiogenesis-related factors in breast cancer cells and found that the mRNA levels of VEGFA and FGF2 were dramatically increased in PCDHB17P overexpression group (**Supplementary Figure 2A**). Next, we collected the conditioned medium (CM) from MDA-MB-231 and MCF-7 cells transfected with PCDHB17P overexpression or sh-PCDHB17P plasmid. Consistent with the mRNA levels, the protein expression of VEGFA and FGF2 was dramatically increased in the CM from PCDHB17P overexpression group and decreased in the PCDHB17P silenced group (**Figure 3B**, **Supplementary Figure 2B**). When exposed the CM to human umbilical vascular endothelial cells (HUVEC) cells, CM from PCDHB17P overexpression breast cancer cells promoted the tube formation ability, whereas the angiogenesis was inhibited by the CM from PCDHB17P-silenced breast cancer cells (**Figures 3C, D**, **Supplementary Figure 2C**). Meanwhile, PCDHB17P overexpression-derived CM significantly promoted the migration and invasion of HUVEC cells, whereas CM from PCDHB17P knockdown breast cancer cells weaken the migratory and invasive abilities of HUVEC cells (**Figures 3E, F**, **Supplementary Figures 2D, E**). Increased VEGFA and CD31 expression was further detected in xenografts infected with PCDHB17 overexpression breast cancer cells (**Figure 3G**). The above mentioned data suggested that PCDHB17P promoted the angiogenesis of breast cancer cells.

### PCDHB17P Competes With miR-145-3p in Breast Cancer Cells

**Figure 4:**
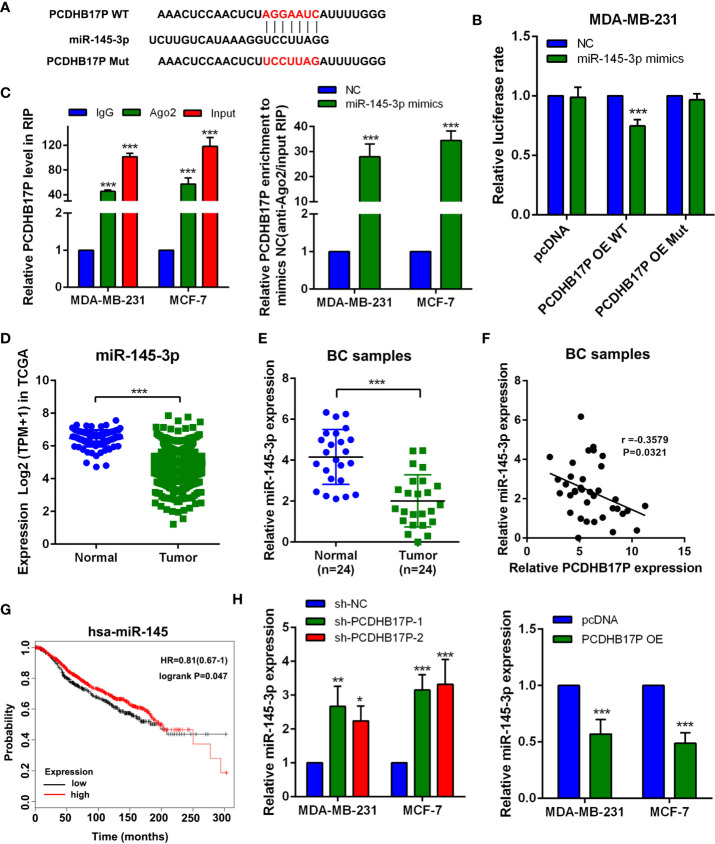
PCDHB17P competes with miR-145-3p in breast cancer cells **(A)** The predicted miR-145-3p binding sites in the PCDHB17P transcript. **(B)** The luciferase activities in MDA-MB-231 cells co-transfected with wild-type (WT) or mutant PCDHB17P plasmid together with miR-145-3p mimic or miR-NC. **(C)** RIP experiments revealed the enrichment of PCDHB17P and miR-145-3p in the Ago2 immunoprecipitation compared with the control IgG precipitation. **(D)**The expression of miR-145-3p were analyzed in breast cancer and normal epithelial tissues by TCGA. **(E)** The expression of miR-145-3p were detected in breast cancer and normal tissues by RT-qPCR. **(F)** Correlation between PCDHB17P and miR-145-3p expression in breast cancer clinical samples. **(G)** Kaplan-Meier analysis of the correlation between miR-145-3p expression and overall survival in TCGA database. **(H)** The expression of miR-145-3p was detected in MDA-MB-231 and MCF-7 cells transfected with PCDHB17P overexpression plasmid or sh-PCDHB17P by RT-qPCR. ^*^ *P* < 0.05, ^**^ *P* < 0.01, ^***^ *P* < 0.001.

lncRNAs could act as ceRNAs to regulate tumor oncogenes or suppressor genes *via* binding with miRNAs. We have confirmed PCDHB17P was mainly located in the cytoplasm. Using LncRNABase and NONCODE online database we found potential binding sites between PCDHB17P and miR-145-3p (**Figure 4A**). Luciferase reporter showed that overexpression of miR-145-3p reduced luciferase activity in PCDHB17P wild-type group, but not the PCDHB17P mutant group (**Figure 4B**). Next, Ago2-RIP assay also demonstrated that the PCDHB17P enrichment was much higher in miR-145-3p overexpression groups than that in the miR-NC group (**Figure 4C**). The expression of miR-145-3p was significantly decreased in breast cancer samples compared with the adjacent normal tissues by TCGA analysis and RT-qPCR assays (**Figures 4D, E** **)**. In addition, the expression of PCDHB17P was negatively correlated with miR-145-3p expression in breast cancer tissues (**Figure 4F**). What’s more, Kaplan-Meier survival assay indicated that miR-145-3p expression in breast cancer was significantly associated with a higher rate of overall survival (**Figure 4G**). Further investigations showed that PCDHB17P negatively affected miR-145-3p expression (**Figure 4H**). All these results revealed that PCDHB17P might modulate miR-145-3p expression *via* acting as a ceRNA.

### miR-145-3p Suppresses Breast Cancer Cells Metastasis and Angiogenesis *In Vitro*

**Figure 5:**
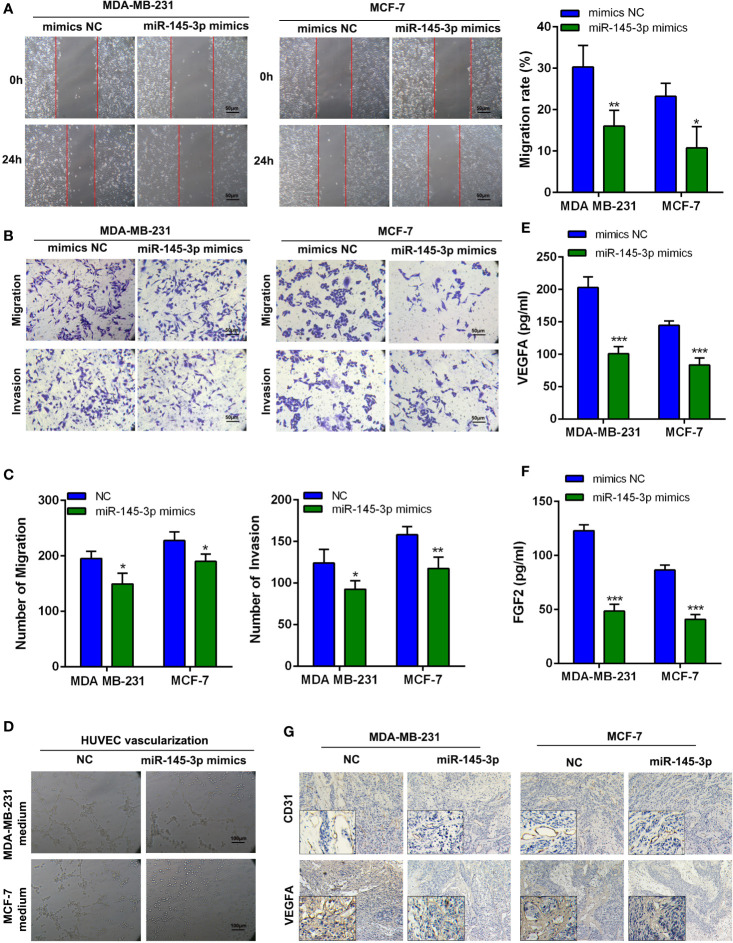
MiR-145-3p promotes breast cancer cells migration, invasion, and angiogenesis *in vitro* **(A)** Wound healing assays were used to assess migration of breast cancer cells transfected with miR-145-3p mimics. **(B, C)** Transwell assay was used to assess migration and invasion of breast cancer cells transfected with miR-145-3p mimics. **(D)** Tube formation of HUVEC cell was detected and results were expressed as number of branches. **(E, F)** The protein level of VEGFA and FGF2 in CM by ELISA assays. **(G)** The expression of VEGFA and CD31 in the xenografts was examined by IHC. ^*^ *P* < 0.05, ^**^ *P* < 0.01, ^***^ *P* < 0.001.

To evaluate the influence of miR-145-3p on breast cancer cells metastasis and angiogenesis, MDA-MB-231 and MCF-7 cells were transfected with miR-145-3p mimics or inhibitor (**Supplementary Figure 3A**) and subjected to Wound Healing, Transwell assays, and tube formation assays. The results revealed that miR-145-3p mimics dramatically inhibited the migration, invasion, and angiogenesis of breast cancer cells, whereas the opposite effects were observed with miR-145-3p inhibitor (**Figures 5A–D**, **Supplementary Figures 3B–G**). In addition, the protein levels of VEGFA and FGF2 were dramatically decreased in the CM from miR-145-3p mimics group and increased in the CM from miR-145-3p inhibitor group (**Figures 5E, F**, **Supplementary Figure 3H**). To further elucidate whether miR-145-3p contributed to metastasis and angiogenesis of breast cancer, we evaluated the expression levels of VEGFA and CD33 by IHC. Compared with tumors derived from NC group, decreased VEGFA and CD31 expression was detected in miR-145-3p mimics group (**Figure 5G**). The data suggested that miR-145-3p suppressed the metastasis and angiogenesis of breast cancer cells.

### MiR-145-3p Suppresses MELK in Breast Cancer Cells

**Figure 6:**
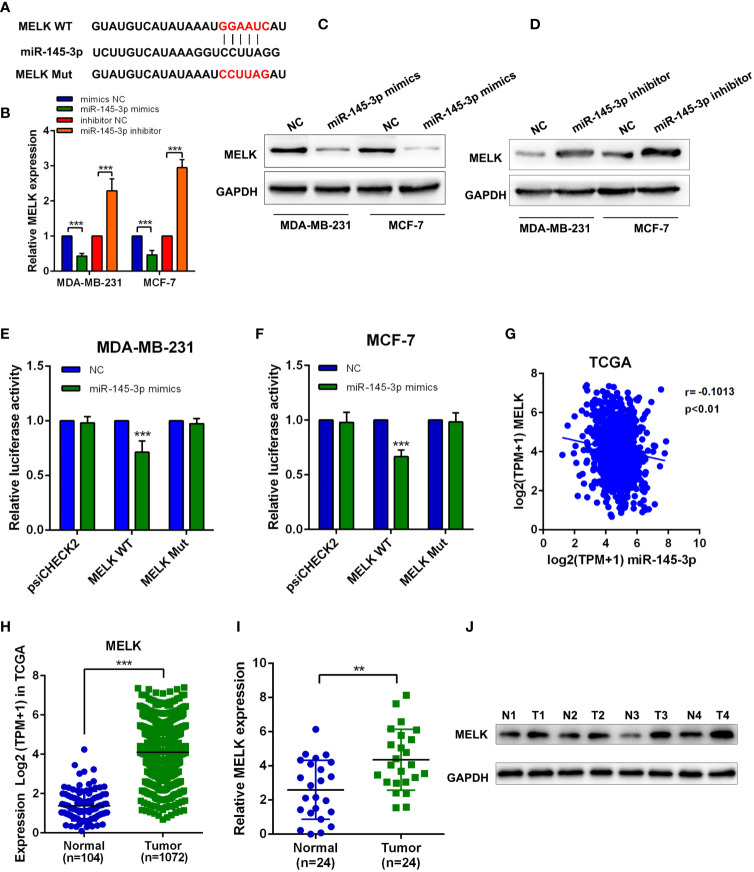
MiR-145-3p suppresses MELK in breast cancer cells **(A)** The predicted miR-145-3p binding sites in the MELK transcript. **(B)** The expression of MELK was detected in MDA-MB-231 and MCF-7 cells transfected with miR-145-3p mimics or miR-145-3p inhibitor by RT-qPCR. **(C, D)** The expression of MELK was detected in MDA-MB-231 and MCF-7 cells transfected with miR-145-3p mimics or miR-145-3p inhibitor by western blot. **(E, F)** The luciferase activities in MDA-MB-231 and MCF-7 cells co-transfected with wild-type (WT) or mutant MELK plasmid together with miR-145-3p mimic or miR-NC **(G)** Correlation between MELK and miR-145-3p expression based on the TCGA analysis. **(H)** MRNA expression level of MELK in breast cancer samples and normal tissues from TCGA database **(I)** MRNA expression level of MELK in breast cancer samples and normal tissues by RT-qPCR assays. **(J)** The protein level of MELK in breast cancer samples and normal tissues from western blotting. ^**^ *P* < 0.01, ^***^ *P* < 0.001.

To further explore the downstream target of miR-145-3p, we applied bioinformatics analysis and found the binding sites between miR-145-3p and MELK (**Figure 6A**). Further experiments showed that miR-145-3p negatively affected MELK expression both at mRNA and protein levels (**Figures 6B–D**). Luciferase reporting experiments revealed that overexpression of miR-145-3p reduced the luciferase activity of MELK wild-type group, but not the MELK mutant group (**Figures 6E, F** **)**. TCGA database analysis showed that the expression of MELK and miR-145-3p was negatively correlated in Breast cancer tissues (**Figure 6G**). MELK level was increased in breast cancer samples compared with that in the normal tissues, as confirmed by the TCGA database and frozen clinical samples (**Figures 6H** **–J**). What’s more, the expression of MELK and PCDHB17P were positively correlated in Breast cancer tissues (**Supplementary Figure 4A**). In addition, when comparing with tumors derived from NC group, tumors from the PCDHB17P overexpression group exhibited increased MELK expression. (**Supplementary Figure 4B**). Therefore, miR-145-3p suppressed MELK expression in breast cancer cells.

### PCDHB17P Promotes MELK-Mediated Metastasis and Angiogenesis Through miR-145-3p Sponging *In Vitro*

**Figure 7:**
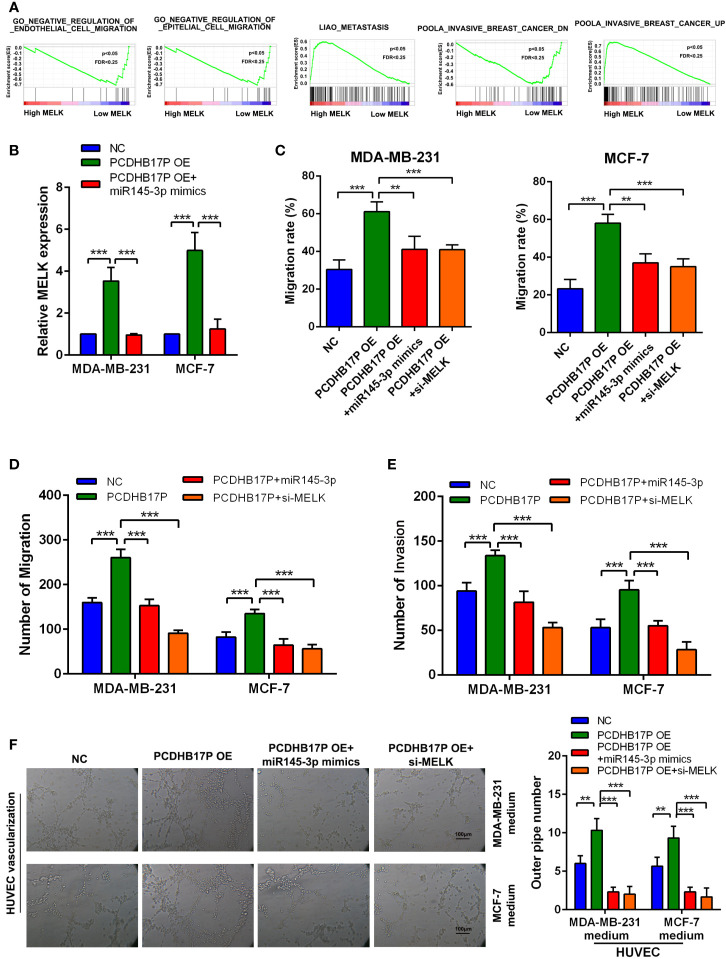
PCDHB17P promotes MELK-mediated metastasis and angiogenesis through miR-145-3p sponging *in vitro* **(A)** GSEA revealed that MELK expression was related to metastasis and angiogenesis in breast cancer. **(B)** RT-qPCR results of the expression of MELK in MDA-MB-231 and MCF-7 cells in response to PCDHB17P overexpression, or PCDHB17P overexpression plus miR-145-3p mimics. **(C–E)** Wound healing and transwell assays were used to assess metastasis of breast cancer cells transfected with PCDHB17P or PCDHB17P simultaneously with miR-145-3p mimics or si-MELK. **(F)** Tube formation of HUVEC cell was detected and results were expressed as number of branches. ^**^ *P* < 0.01, ^***^ *P* < 0.001.

GSEA based on the breast cancer TCGA datasets showed MELK expression was related to metastasis and angiogenesis (**Figure 7A**). PCDHB17P positively increased MELK expression, while miR-145-3p mimics partially attenuated the effects of PCDHB17P on the expression levels of MELK (**Figure 7B**). To confirmed whether PCDHB17P modulate breast cancer progression in a MELK-dependent manner, we transfected breast cancer cells stably overexpressing PCDHB17P with miR-145-3p mimics or si-MELK. *In vitro* function experiments demonstrated that MELK knockdown or miR-145-3p mimics partially rescued the stimulative effects of PCDHB17P overexpression on breast cancer cells migration, invasion, and angiogenesis (**Figures 7C–F**, **Supplementary Figures 5A, B**). These findings demonstrated that PCDHB17P was an oncogenic lncRNA that promotes breast cancer cells migration and angiogenesis *via* the PCDHB17P/miR-145-3p/MELK axis.

### PCDHB17P Is Feedback Transcriptionally Regulated by NF-κB

**Figure 8:**
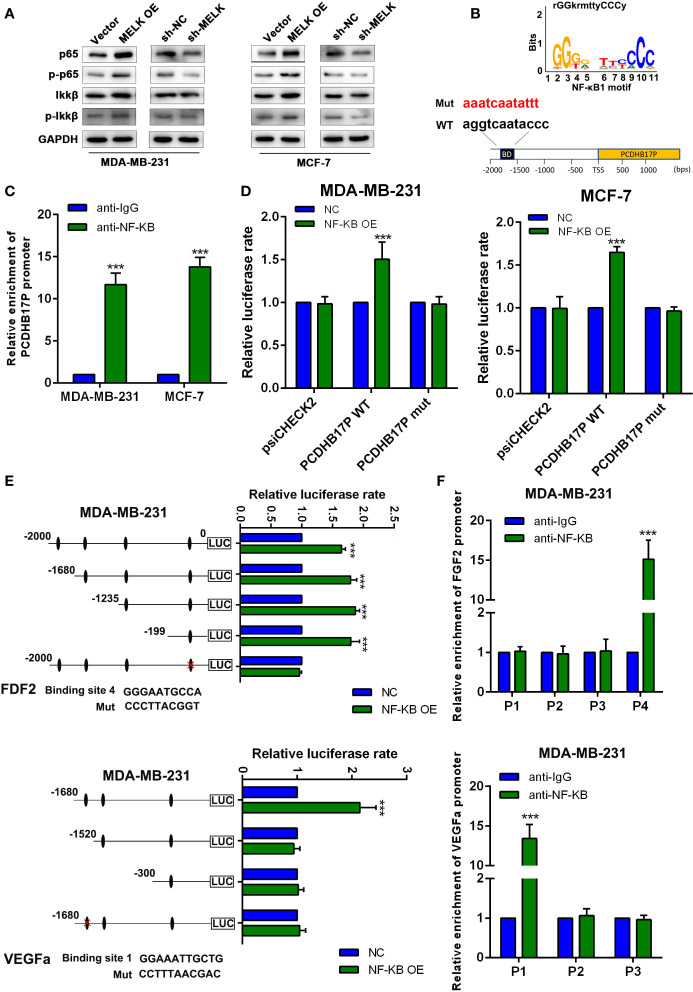
PCDHB17P is feedback transcriptionally regulated by MELK. **(A)** The expression of P65, p-P65, IKKβ and p-IKKβ were detected in MDA-MB-231 and MCF-7 cells transfected with MELK overexpression by western blotting. **(B)** The binding motif of NF-κB from JASPAR tool. **(C)** ChIP assay demonstrating the direct binding of NF-κB to the PCDHB17P promoter in MDA-MB-231 and MCF-7 cells. **(D)** Luciferase reporter assay was performed in MDA-MB-231 and MCF-7 cells co-transfected NF-κB OE or pcDNA with wild-type or mutated PCDHB17P. **(E)** Luciferase reporter assays were used to locate the binding sequences of NF-κB to the FGF2 and VEGFA promoters in MDA-MB-231 cells. **(F)** ChIP assay demonstrating the direct binding of NF-κB to the FGF2 and VEGFA promoters in MDA-MB-231 cells. The y-axis represents the relative enrichment with the anti-NF-κB antibody compared to that with the IgG control. ***P < 0.001.

Radoslav has proved MELK could activate NF-κB signaling pathway (16), which inspires us whether PCDHB17P is feedback transcriptionally regulated by NF-κB. In consistent with previous study, compared with the NC group, the expression of NF-κB in MELK overexpression group increased (**Figure 8A**). Interestingly, we found that the transcription factor NF-κB might binds with the PCDHB17P promoter region by JASPAR tool (**Figure 8B**). We knocked down and upregulated NF-κB expression in MDA-MB-231 and MCF-7 cells (**Supplementary Figure 6A**), and RT-qPCR assays revealed that the expression of PCDHB17P was increased in MDA-MB-231 and MCF-7 cells transfected with NF-κB overexpression plasmid (**Supplementary Figure 6B**). ChIP analysis revealed that PCDHB17P were abundant in the binding complex of NF-κB (**Figure 8C**). Luciferase reporter showed that overexpression of NF-κB increased the luciferase activity of the wild-type PCDHB17P reporter gene, but not the PCDHB17P mutant vector (**Figure 8D**). What’s more, we found four potential binding sites of NF-κB on the FGF2 promoter and three binding sites on the VEGFA promoter, the direct binding of NF-κB to the FGF2 and VEGFA promoters were validated by luciferase activity assays and ChIP (**Figures 8E, F** **)**. In addition, RT-qPCR assays revealed that the expression of VEGFA and FGF2 were increased in MDA-MB-231 and MCF-7 cells transfected with NF-κB overexpression plasmid (**Supplementary Figure 6C**). Hence, these results from our study revealed that PCDHB17P/miR-145-3p/MELK/NF-κB formed a positive feedback loop in breast cancer cells.

## Discussion

Metastasis and angiogenesis are typical characteristics of breast cancer, which lead to poor prognosis, frequent recurrence, and pronounced invasiveness (17). Accumulating evidence has revealed that lncRNAs play an important role in the carcinogenesis and progression of breast cancer by influencing critical events such as cell proliferation, metastasis, and angiogenesis (18). In the present study, we firstly figured out a novel lncRNA PCDHB17P, which was highly expressed in breast cancer tissues and also associated with poor prognosis. Experimental results confirmed that overexpression of PCDHB17P remarkably promoted angiogenesis and metastasis both *in vitro* and *in vivo*. These findings demonstrated that PCDHB17P played an important role in breast cancer progression and inspired us to conduct further mechanistic research.

In recent years, accumulated evidence shows that there is a novel regulatory mechanism between lncRNAs and miRNAs. LncRNAs may act as ceRNAs, which were first proposed in 2011 (19), to negatively regulate the miRNA expression thus playing a crucial role in ceRNA networks in human cancers. For instance, lncRNA NONHSAT101069 functioned as a ceRNA *via* sponging miR-129-5p, promoted epirubicin resistance, migration, and invasion of breast cancer cells (20). LINC00963 promoted tumorigenesis and radioresistance in breast cancer cells by sponging miR-324-3p (21). In our study, FISH and subcellular fractionation both revealed that PCDHB17P was largely located in the cytoplasm. Then, bioinformatics analysis, RIP, and luciferase assays verified that miR-145-3p can act as the direct target for PCDHB17P. It was reported that miR-145-3p is down-regulated in non-small cell lung cancer, and inhibited cell migration and invasion by targeting PDK1 *via* the mTOR signaling pathway (22). Our data showed that miR-145-3p was expressed at low levels in breast cancer tissues and related to survival rates in breast cancer patients. In addition, miR-145-3p inhibitor remarkably promoted tumor-induced angiogenesis and metastasis *in vitro* and *vivo*.

According to TCGA database analysis, PCDHB17P expression was positively correlated with MELK. In addition, the 3′-UTR region of MELK contained potential binding sites for miR-145-3p. Then, we performed a series of experiments and verified the hypothesis that miR-145-3p was a key mediator in the PCDHB17P/miR-145-3p/MELK axis in breast cancer. The role of maternal embryonic leucine zipper kinase (MELK) in cancer is varied, including proliferation, apoptosis, epithelial transformation, and metastasis. Recent studies indicated that MELK was highly expressed in several human cancers, including breast, prostate cancer, gastric cancer, and lung cancer (23). MELK is also associated with poor patient survival in breast cancer (24). In this study, we revealed that MELK was expressed at high levels in breast cancer tissues, which was consistent with previous researches. In addition, we referred to GSEA and found that MELK expression was related to metastasis and angiogenesis, rescue assays confirmed that the PCDHB17P/miR-145-3p/MELK axis was involved in the migration, invasion, and angiogenesis of breast cancer.

The activation of NF-κB stimulates tumor cell growth, inhibits tumor cells apoptosis, and enhances tumor invasion, metastasis, and angiogenesis (25). In a previous study, MELK has been proven to promote melanoma growth by activating NF-κB pathway activity (16). Consistently, we demonstrated that overexpression of MELK promoted the expression of NF-κB. Multiple studies have demonstrated that lncRNAs can be regulated by transcription factors in the cell nucleus (26, 27). According to the prediction tools, there were potential binding sites of NF-κB on the PCDHB17P promoter. Based on ChIP and luciferase activity assays, we verified the hypothesis and located the binding sequences in the PCDHB17P promoter region. Furthermore, we also demonstrated that overexpression of NF-κB promoted the expression of PCDHB17P. Moreover, VEGFA and FGF2, two pro-angiogenesis factors were also transcriptionally stimulated by NF-κB, thus promoting the angiogenesis of breast cancer. These findings revealed a positive feedback loop (MELK/PCDHB17P/miR-145-3p/MELK/NF-κB), which facilitated the metastasis and angiogenesis of breast cancer.

In summary, the present study firstly revealed that PCDHB17P was upregulated in breast cancer tissues and cell lines, MELK mediated PCDHB17P-induced promotion on breast cancer metastasis and angiogenesis by sponging miR-145-3p. The evidence provided by this study supported the instance that the PCDHB17P/miR-145-3p/MELK/NF-κB axis is implicated in the metastasis and angiogenesis of breast cancer and may be considered as a potential target for the breast cancer therapies in the future.

## Data Availability Statement

The original contributions presented in the study are included in the article/**Supplementary Material**. Further inquiries can be directed to the corresponding authors.

## Author Contributions

LZ, Y-JZ, and BW performed the study designing, experiments, and manuscript writing. LY and Y-JZ: experiments, analysis, and interpretation of data. L-DS and LT: collection, interpretation, and analysis of data. J-DW and TC: interpretation of data and manuscript revision. All authors contributed to the article and approved the submitted version.

## Funding

This study was supported by Young Elite Scientists Sponsorship Program by China Association for Science and Technology (17-JCJQ-QT-029), Researching Funding of Medical Education Association (2016001), and Science Foundation of the PLA General Hospital (2018FC-WJFWZX-2-09).

## Conflict of Interest

The authors declare that the research was conducted in the absence of any commercial or financial relationships that could be construed as a potential conflict of interest.

## Abbreviations

lncRNAs, long non-coding RNAs; ceRNA, competing endogenous RNA; EMT, epithelial-mesenchymal transition; miRNAs, microRNAs; HCC, hepatocellular carcinoma; BC, breast cancer; H&E, hematoxylin and eosin; GSEA, gene set enrichment analysis.
