# Supplementary material for: PCDHB17P/miR-145-3p/MELK/NF-κB Feedback Loop Promotes Metastasis and Angiogenesis of Breast Cancer
Source: Front Oncol. 2021 Jul 19;11:660307. doi: 10.3389/fonc.2021.660307 (PMC8327775; doi:10.3389/fonc.2021.660307)
Supplement: Supplementary file 1 [file DataSheet_1.doc]

**PCDHB17P/miR-145-3p/MELK/NF-κB feedback loop promotes metastasis and angiogenesis of breast cancer**

Li Zhu^1,*^, Yan-Jun Zhang^1,*^, Bin Wang^2,*^, Li Yang^3^, Yi-Qiong Zheng^1^, Lin-De Sun^1^, Lin Tian^1^, Tao Chen^4,#^, Jian-Dong Wang^1,#^

**Supplementary Table 1. Sequences of shRNA Against Specific Targets**

| sh-PCDHB17P-1 | 5’-3’ | GGGAATTTGCTCCTAAAAGAAAA |
| --- | --- | --- |
| sh-PCDHB17P-2 | 5’-3’ | GGGACTTCATTTCCTTTGAAAAT |
| si-MELK | 5’-3’ | TTGCTGTTTGATGAATATCATAA |
| si-P65 | 5’-3’ | AGGACATTGAGGTGTATTTCACG |

**Supplementary Table 2. Sequences of PCR primers used in this study**

| PCDHB17P | Forward(5’-3’) | TTCTTGGGTGAAGGGACTGG |
| --- | --- | --- |
|  | Reverse(5’-3’) | TCACGACCTGTTGTCAGATTCC |
| MELK | Forward(5’-3’) | AAGAAGGCTCGGGGAAAACC |
|  | Reverse(5’-3’) | GCAGGTGTTCTGCATAAGGC |
| IL-8 | Forward(5’-3’) | ACTCCAAACCTTTCCACCCC |
|  | Reverse(5’-3’) | ATGAATTCTCAGCCCTCTTCAA |
| VEGFA | Forward(5’-3’) | TGCCCGCTGCTGTCTAATG |
|  | Reverse(5’-3’) | GCGAGTCTGTGTTTTTGCAGG |
| FGF2 | Forward(5’-3’) | GCAAAAACGGGGGCTTCTTC |
|  | Reverse(5’-3’) | AACGGTTAGCACACACTCCT |
| PDGFC | Forward(5’-3’) | ATGAGCCTCTTCGGGCTTCT |
|  | Reverse(5’-3’) | TACTCAGGTTGGATTCCGCC |
| PDGFD | Forward(5’-3’) | GCACCGGCTCATCTTTGTCT |
|  | Reverse(5’-3’) | GATTGCTCTCATCTCGCCTG |
| AGGF1 | Forward(5’-3’) | TGGAGGAATGAAAACGCCGA |
|  | Reverse(5’-3’) | TGAGGATGGTTTGCCTGTCC |
| miR-145-3p | Forward(5’-3’) | AGGGATTCCTGGAAATACTGT |

| **Supplementary Table 3 . Correlation of clinicopathological characteristics and gene expression in the patients** | | | | | |
| --- | --- | --- | --- | --- | --- |
| Variables | | No. of patients (n=819) | expression | | p-value |
|  |  |  | Low(409) | High(410) |  |
| Age (yr) | |  |  |  | 0.005 |
|  | ＜45 | 130 | 80 | 50 |  |
|  | ≥45 | 689 | 329 | 360 |  |
| Tumor invasion (T) | |  |  |  | . |
|  | T1 | 213 | 109 | 104 | 0.604 |
|  | T2 | 487 | 247 | 240 |  |
|  | T3 | 90 | 39 | 51 |  |
|  | T4 | 29 | 14 | 15 |  |
|  | T1+T2 | 700 | 356 | 344 | 0.24 |
|  | T3+T4 | 119 | 53 | 66 |  |
| Clinical stage | |  |  |  |  |
|  | Stage I | 146 | 75 | 71 | 0.357 |
|  | Stage II | 473 | 240 | 233 |  |
|  | Stage III | 183 | 89 | 94 |  |
|  | Stage IV | 17 | 5 | 12 |  |
|  | Stage I+II | 619 | 315 | 304 | 0.382 |
|  | Stage III+IV | 200 | 94 | 106 |  |
| Lymph nodes metastasis | |  |  |  | 0.032 |
|  | No | 555 | 292 | 263 |  |
|  | Yes | 264 | 117 | 147 |  |
| Distant metastasitc | |  |  |  | 0.143 |
|  | No | 802 | 404 | 398 |  |
|  | Yes | 17 | 5 | 12 |  |
| * p<0.05. Pearson's Chi-squared test and Fisher's Exact Test | | | | | |

**Supplementary Figure 1. PCDHB17P promotes metastasis of Breast cancer cells**


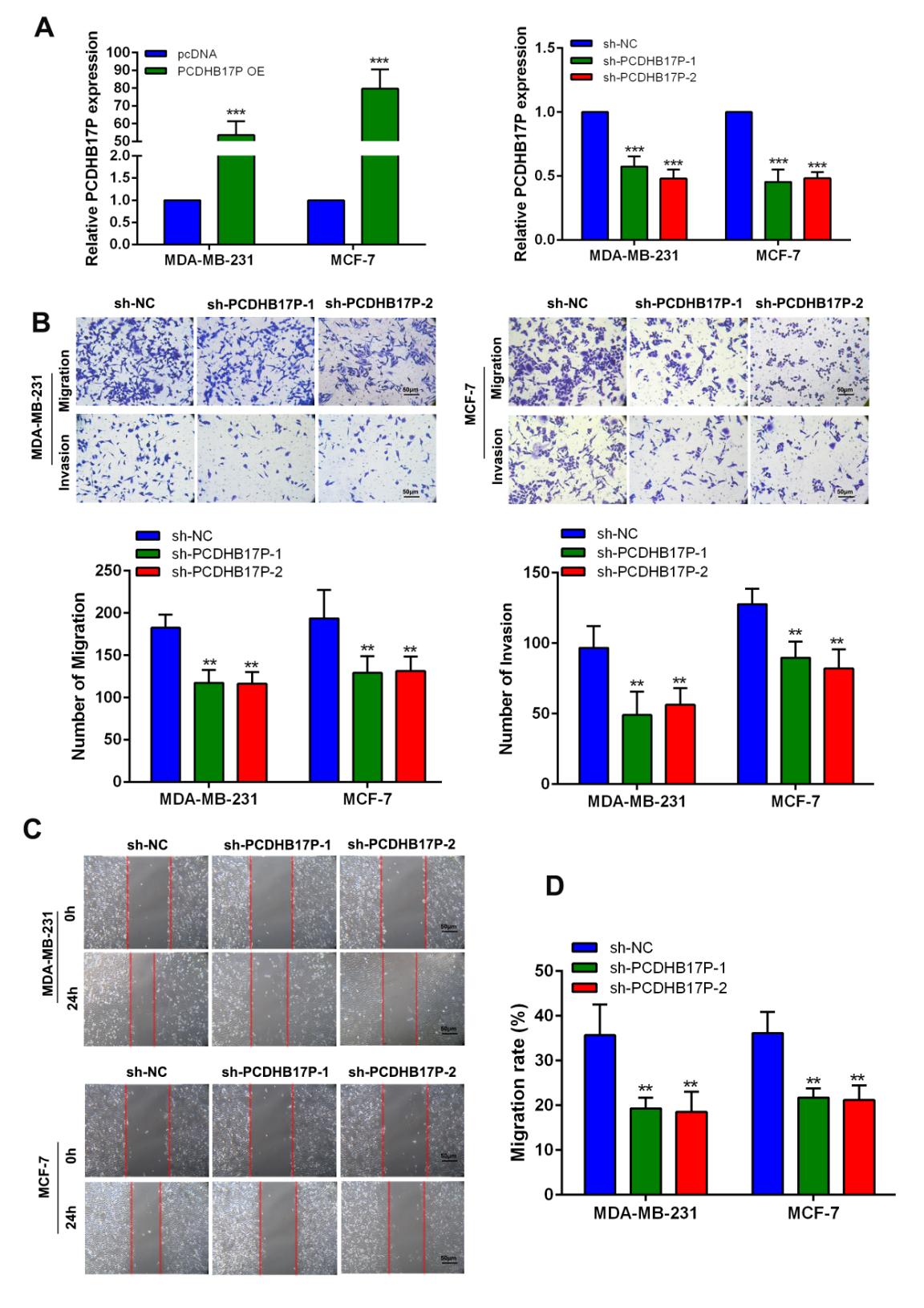


(A) The expression of PCDHB17P was detected in MDA-MB-231 and MCF-7 cells stably transfected with PCDHB17P OE or sh-PCDHB17P by RT-qPCR. (B-D) Influence of PCDHB17P knockdown on cell migration and invasion of MDA-MB-231 and MCF-7 cells by the transwell and wound healing assays. ^*^*P*<0.05, ^**^*P*<0.01, ^***^*P*<0.001.

**Supplementary Figure 2. PCDHB17P promotes** **angiogenesis of BRCA cells**

**
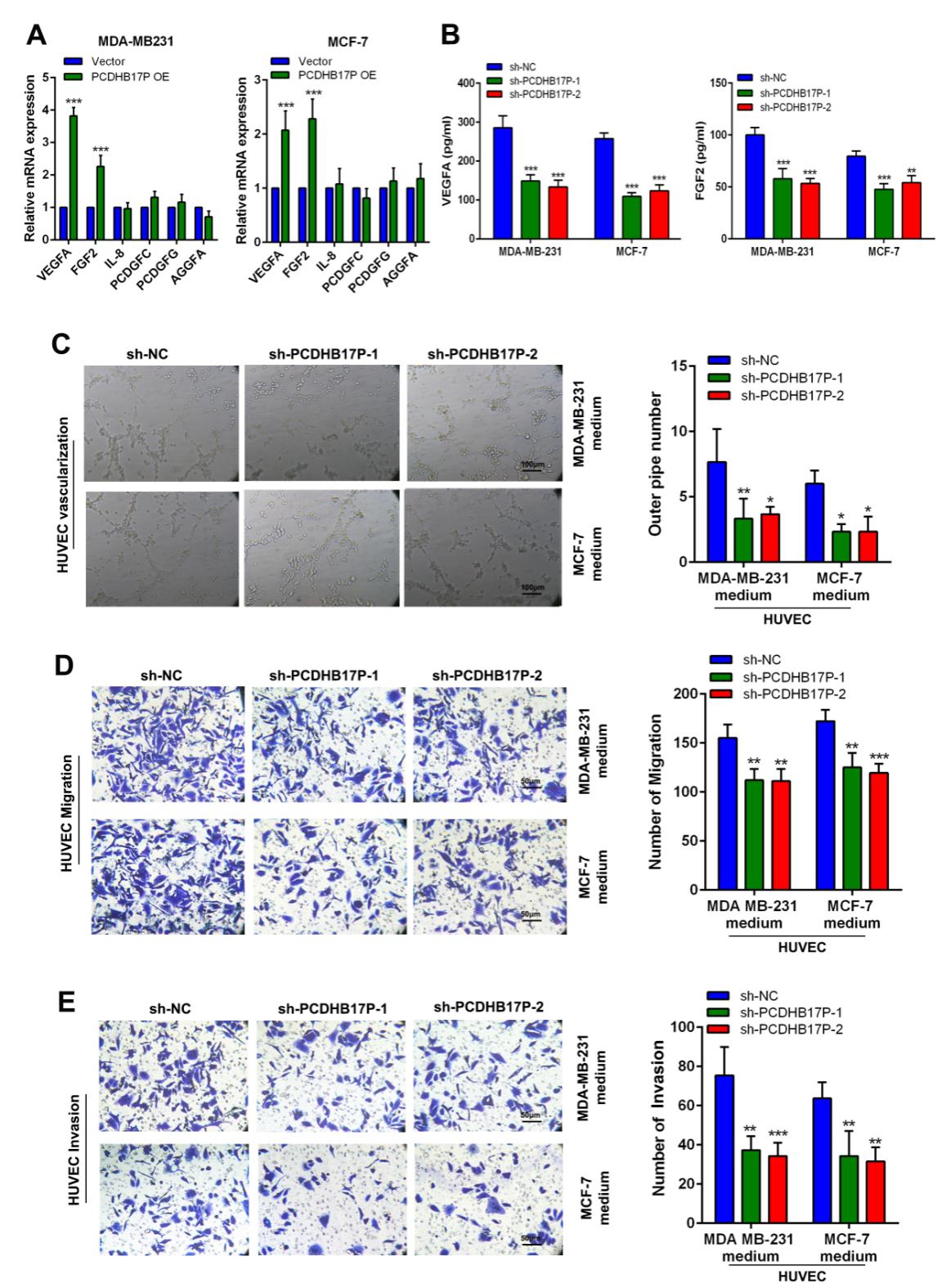
**

1. MRNA levels of angiogenesis-related factors in MDA-MB-231 and MCF-7 cells supernatant. (B) The protein level of VEGFA and FGF2 in CM by ELISA assays. (C) Tube formation of HUVEC cell was detected and results were expressed as number of branches. (D-E) HUVEC cell migration and invasion were determined by transwell assay.  ^*^*P*<0.05, ^**^*P*<0.01, ^***^*P*<0.001.

**Supplementary Figure3.** **MiR-145-3p promotes breast cancer cells migration, invasion and angiogenesis in vitro**


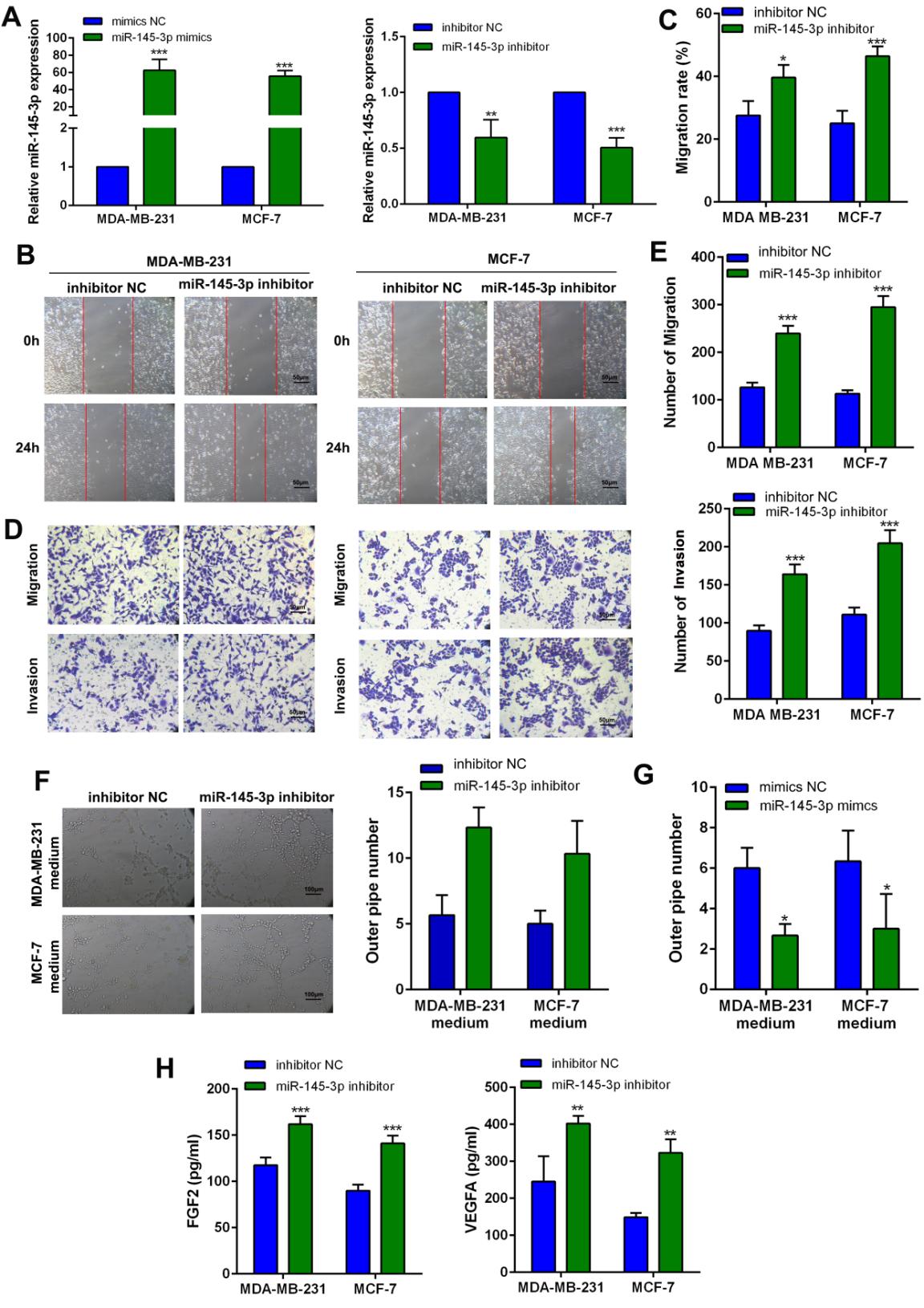


1. The level of miR-211-5p was detected in MDA-MB-231 and CAL-27 cells transfected with miR-211-5p mimics or miR-211-5p inhibitor. (B-C) Wound healing assays were used to assess migration of breast cancer cells transfected with miR-145-3p inhibitor. (D-E) Transwell assay was used to assess migration and invasion of breast cancer cells transfected with miR-145-3p inhibitor. (F-G)Tube formation of HUVEC cell was detected and results were expressed as number of branches. (H) The protein level of VEGFA and FGF2 in CM by ELISA assays. ^*^*P*<0.05, ^**^*P*<0.01, ^***^*P*<0.001.

**Supplementary Figure4. The expression of PCDHB17P was possitively correlated with MELK in Breast cancer**

**
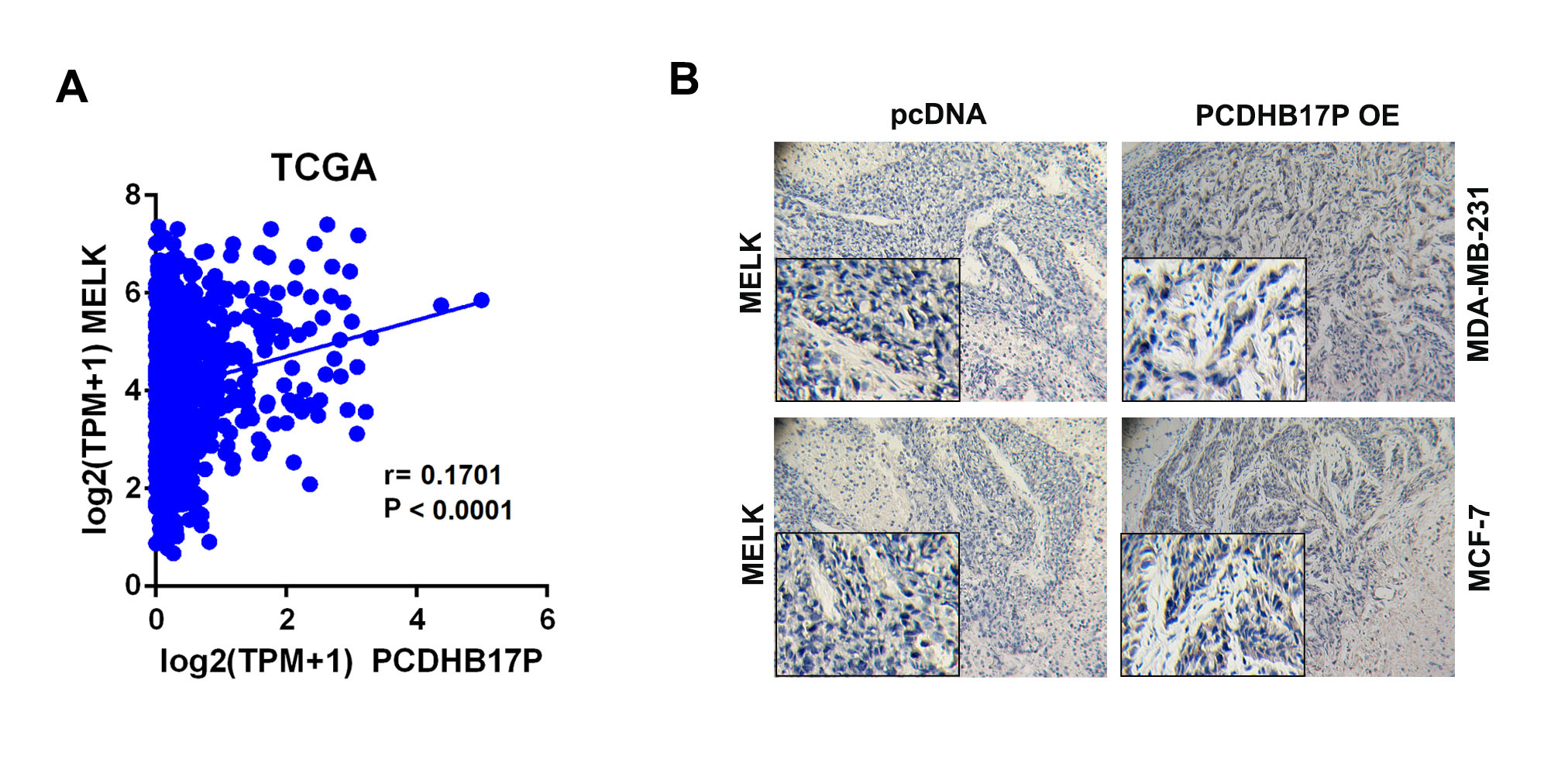
**

1. Correlation between MELK and PCDHB17P expression based on the TCGA analysis.(B) The expression of MELK in the xenografts was examined by IHC.^*^*P*<0.05, ^**^*P*<0.01, ^***^*P*<0.001.

**Supplementary Figure5. PCDHB17P promotes MELK mediated metastasis and angiogenesis through** **miR-145-3p sponging in vitro**


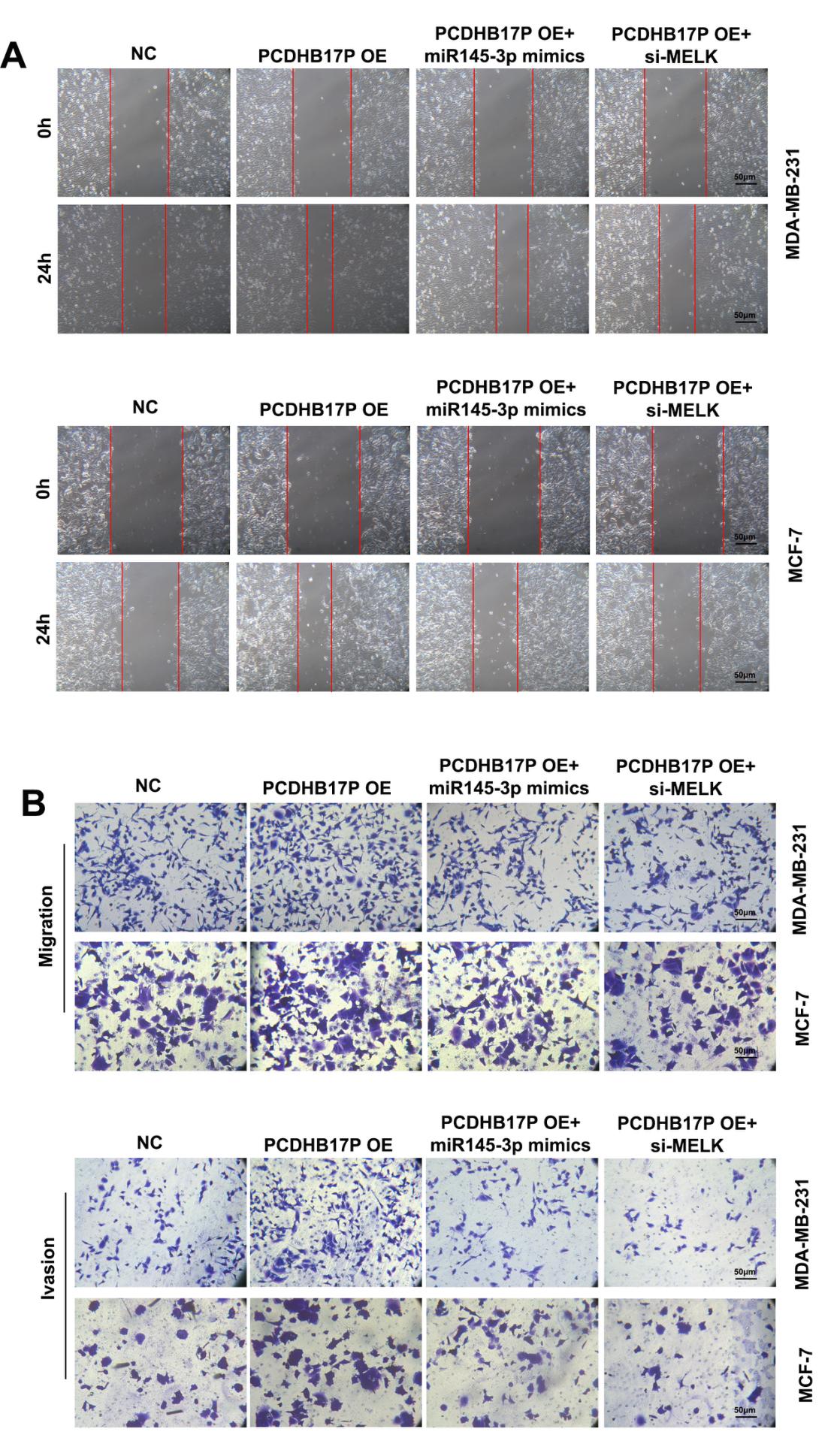


(A-B) Wound healing and transwell assays were used to assess metastasis of breast cancer cells transfected with PCDHB17P or PCDHB17P simultaneously with miR-145-3p mimics or si-MELK.  ^*^*P*<0.05, ^**^*P*<0.01, ^***^*P*<0.001.

**Supplementary Figure6.** **PCDHB17P is feedback transcriptionaly regulated by MELK**


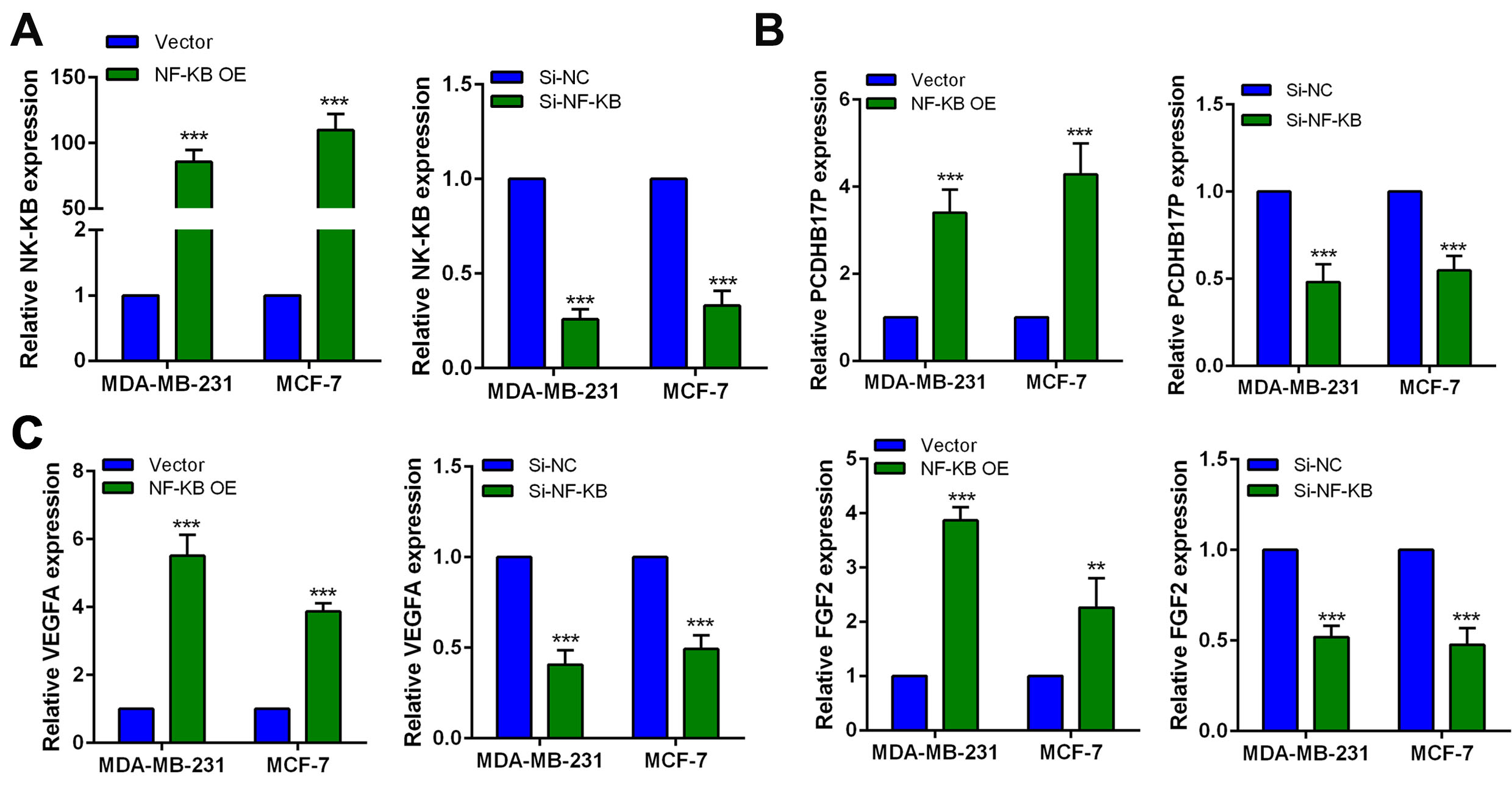


(A) The expression of NF-κB was detected in MDA-MB-231 and CAL-27 cells transfected with Si-NF-κB or NF-κB OE by RT-qPCR. (B) The expression of PCDHB17P was detected in MDA-MB-231 and CAL-27 cells transfected with Si-NF-κB or NF-κB OE by RT-qPCR. (C) The expression of VEGFA and FGF2 were detected in MDA-MB-231 and CAL-27 cells transfected with Si-NF-κB or NF-κB OE by RT-qPCR. ^*^*P*<0.05, ^**^*P*<0.01, ^***^*P*<0.001.
